# Anticholinesterase and antioxidant potentials of *Nonea micrantha* Bioss. & Reut along with GC-MS analysis

**DOI:** 10.1186/s12906-017-2004-9

**Published:** 2017-11-23

**Authors:** Muhammad Imran, Farhat Ullah, Muhammad Ayaz, Abdul Sadiq, Muhammad Raza Shah, Muhammad Saeed Jan, Farman Ullah

**Affiliations:** 1 Department of Pharmacy, University of Malakand, Chakdara, Dir Pakistan; 2 HEJ Research Institute of Chemistry, International Center for Chemical and Biological Sciences, Karachi University, Karachi, 74200 Pakistan; 3 Kohat University of Science and Technology, Kohat, Pakistan

**Keywords:** Acetylcholinesterase, Butyrylcholinesterase, DPPH, ABTS, TRP, *N. micrantha*

## Abstract

**Background:**

*Nonea micrantha Boiss. & Reut* ***.*** being an unexplored member of Boraginaceae was investigated for GC/MS analysis, acetylcholinesterase (AChE), butyrylcholinesterase (BChE) inhibitory and antioxidant activities in an attempt to find its effectiveness in neurological disorders.

**Methods:**

The AChE and BChE inhibitory activities of crude methanolic extract (Nm.Cr), subsequent fractions; *n*-hexane (Nm.Hex), chloroform (Nm.Cf), ethyl acetate (Nm.EtAc), aqueous (Nm.Aq) and crude saponins (Nm.Sp) from *N. micrantha* were conducted using Ellman’s assay. The antioxidant activity of the plant samples using DPPH and ABTS free radical scavenging potential following quantitative spectrophotometric and qualitative TLC method were also studied. Moreover the total reducing power (TRP) of all the samples was also figured out.

**Results:**

The GC/Ms analysis confirmed that the plant is rich in bioactive molecules. Among different fractions, Nm.Hex, Nm.EtAc and Nm.Cf exhibited highest AChE inhibitory activities causing 75.51 ± 0.73, 68.54 ± 0.59 and 63.48 ± 0.59% enzyme inhibition respectively and IC_50_ of 44, 100 and 144 μg/mL respectively. In BChE inhibiton assay, Nm.Aq, Nm.Sp and Nm.Cr showed highest activity causing 83.49 ± 0.27, 81.49 ± 0.89 and 75.31 ± 0.56% enzyme inhibition with IC_50_ of 90, 110 and 44 μg/mL respectively. In DPPH assay, Nm.Aq, Nm.Cf, Nm.Hex and Nm.Cr were most potent exhibiting IC_50_ values of 3, 5, 93 and 120 μg/ml respectively. In ABTS assay Nm.EtAc, Nm.Aq, Nm.Sp and Nm.Cr showed IC_50_ values of 60, 95, 100 and 150 μg/mL respectively. Likewise ABTS inhibition was most prominent for Nm.Sp, Nm.EtAc and Nm.Aq causing 78.26 ± 0.49, 67.67 ± 0.73 and 63.58 ± 0.45% inhibition respectively at 1 mg/mL. These results were further confirmed by qualitative screening using DPPH and ABTS staining.

**Conclusions:**

Our anticholinesterase and antioxidant results signify the *N. micrantha* as a potential source of natural bioactive compounds. Moreover isolation of natural bioactive compounds from this plant may lead to novel drug candidates against neurodegenerative disorders.

## Background

Alzheimer’s disease (AD) is a chronic neurodegenerative disorder characterized by loss of cognitive ability, severe behavioral abnormalities and ultimately leads to death. AD is the most common cause of dementia especially among the elder people. There are presently 24.3 million estimated AD patients all over the world, with 4.6 million new cases of dementia every year [1]. This disease is associated with a cholinergic deficit in the post-mortem brain characterized by a significant decrease in acetylcholine (ACh) amount [2, 3]. ACh, a neurotransmitter inhibited primarily by acetylcholinesterase (AChE) and butyrylcholinesterase (BChE), plays a role in the pathology of AD [4, 5]. Therefore, AChE and BChE inhibitors have become the most useful alternatives in the treatment of AD. Drugs as eserine, tacrine, donepezil, rivastigmine, and galanthamine have been approved for the treatment of AD. However, these drugs are known to have limitations for clinical use due to their short half lives and antagonistic side effects [6, 7]. Therefore, the search for new AChEIs and BChEIs with higher efficacy and safety from alternative sources like natural products is the focus of multiple investigators [8].

On daily basis, various complex redox reactions in our body results in the production of reactive oxygen species (ROS) i.e., Hydroxyl radical (OH^•^), super oxide radicals (O_2_^•□^), hydrogen peroxide (H_2_O_2_) and singlet oxygen (O^•^) [9]. The harmful effect of ROS are diminished by certain enzymes in our body i.e., catalase, glutathione peroxidase and superoxide dismutase. If the level of these enzymes gets decreased from that of free radicals, it leads to oxidative stress and eventually certain chronic disorders [10].

Free radicals contribute to more than hundred disorders in humans including atherosclerosis, arthritis, ischemia reperfusion injury of many tissues, central nervous system injury, gastritis, cancer and AIDS [11]. Antioxidants are those substances which may protect cells from the damage caused by unstable molecules of free radicals. Antioxidants interact with free radicals and stabilize them and thus prevent free radicals mediated damages in the body [12]. To counteract the action of free radicals various synthetic antioxidant are being used i.e., Butylated hydroxytoluene (BHT), butylated hyroxyanisole (BHA), and tertiary butyl hydroquinone (TBHQ) but unfortunately these antioxidants are associated with various toxic effect [13, 14]. Various synthetic compounds have also been reported to possess anticholinesterase along with antioxidant potentials [15]. Therefore the natural products, being an alternative option and rich source of antioxidants, have grabbed the focus of scientists. In technologically advanced world, hazards and unexpected side effects that result from the use of synthetic drugs have compelled to investigate plants for the safe and enhanced medicinal values. Majority of herbal medicines do not have such effects and are preferred due to presence of various useful compounds [16].

Plants contain a wide variety of free radical scavenging compounds like phenolic compounds, vitamins, terpenoids, nitrogenous compounds and some other endogenous metabolites with strong antioxidant activity [17]. In recent years, there has been a growing interest in finding natural antioxidants in plants because they inhibit oxidative damage and may consequently prevent aging and neurodegenerative diseases [18]. Medicinal plants have long been used to treat cognitive memory dysfunction symptoms and as possible sources for the discovery of novel antioxidant molecules [19, 20].

Family boraginaceae consists of 156 genera and 2500 species. Traditionally various species of boraginaceae are used in skin diseases, sore throat, gummosis, toothache, hepatic pain, stomach complaints, inflammation, bellyache, as diuretics and against anemia [21, 22]. The antioxidant activity of most of the species of family boraginaceae has been reported with prominent results [23]. Polyphenolic compounds have also been isolated from various species of this family with strong antioxidant activity [24, 25]. In spite of isolation of polyphenolic compounds from various species of boraginaceae, reported antioxidant potentials and ethno medicinal uses, still no research activity has been reported on antioxidant and anticholinesterase potentials of *N. micrantha*. So this study is designed to investigate the anticholinesterase and antioxidant potentials of Nm.Cr, its subsequent fractions and Nm.Sp of *N. micrantha*.

## Methods

### Plant collection

*N. micrantha* whole plant was collected in May 2013 from the hills of Dir Lower, Khyber Pakhtunkhwa, Pakistan. The plant was identified by plant taxonomist, Dr. Ali Hazrat, Department of Botany, SBBU, Dir Upper (KPK) and deposited with voucher number 1021MI/SBBU in herbarium of aforementioned university.

### Extraction

The plant material was washed carefully with tap water and dried under shade at room temperature for 2 weeks. The shade dried (3 kg) parts of the plant were grinded properly and soaked in 80% methanol with occasional shaking. After 15 days, the whole suspension was filtered through muslin cloth. Thereafter the filtrate was concentrated under reduced pressure at 40 °C using rotary evaporator (Heidolph, Germany) [26, 27]. A residue of deep green color (Nm.Cr) weighing 160 g with a percent yield of 5.33% was obtained.

### Fractionation

Crude methanolic extract (140 g) was suspended in 500 mL of distilled water and was consequently partitioned with *n*-hexane (3 × 500 mL), chloroform (3 × 500 mL), ethyl acetate (3 × 500 mL), using separating funnels [28, 29]. Finally Nm.Hex 10 g (7.14%), Nm.Cf 13 g (9.28%), Nm.EtAc 9 g (6.42%) and Nm.Aq 16 g (11.4%) were obtained.

### Extraction of crude saponins

Plant powder material (60 g) was taken in a conical flask. Added 100 mL of 20% ethanol to it and was heated for 4 h at 55 °C with constant shaking in a water bath. Then this mixture was filtered and added 200 mL of 20% ethanol to it. The volume of the extracting liquid was reduced to 40 mL with the help of water bath and was transferred to separating funnel. Then 20 mL of diethyl ether was added with vigorous shaking until two layers were formed. The organic layer was discarded and 60 mL of *n*-butanol was added to the aqueous fraction in a separating funnel. The combined aqueous butanol mixture was washed with 5% NaCl solution several times for removal of impurities. The solvents were evaporated with the help of water bath leaving 7 g of crude saponins (11.66%) [30, 31].

### Gas chromatography (GC) analysis

Nm.Cr was analyzed through Agilent gas chromatograph (Agilent Technologies, USA) having HHP-5MS 5% phenylmethylsiloxane capillary column (30 m × 0.25 mm × 0.25 μm film thickness; Restek, Bellefonte, PA) connected to FID detector. Initially, the oven was maintained at 70 °C for 1 min. Then its temperature was raised at the rate of 6 °C/min to 180 °C for 5 min and finally at the rate of 5 °C/ min to 280 °C for 20 min. The detector and injector were maintained at 290 °C and 220 °C respectively. Helium was employed as carrier gas and its flow was kept as 1 ml/min, and diluted samples (1/1000 in *n*-pentane, *v*/v) of 1.0 μl were injected manually in the splitless mode.

### Gas chromatography/mass spectrometry (GC/MS) analysis

GC/MS analysis of the Nm.Cr was carried with Agilent gas chromatograph (Agilent Technologies, USA) with a HHP-5MS 5% phenylmethylsiloxane capillary column (30 m × 0.25 mm × 0.25 μm film thickness; Restek, Bellefonte, PA) connected with Agilent HP-5973 mass selective detector in the electron impact mode (Ionization energy: 70 eV). The apparatus was operated under the same conditions as mentioned earlier. The detected compounds were identified by comparing their retention times with those of authentic compounds and the spectral data obtained from the Wiley and NIST libraries, as well as comparisons of the fragmentation pattern of the mass spectra with data published in the literature. Each determination was carried out in duplicate [32, 33].

### Anticholinesterase assays

Acetylcholinesterase (AChE) from Electric eel (Sigma-Aldrich, USA), and Butyrylcholinesterase (BChE) from equine serum (Sigma-Aldrich, USA), were used to investigate the enzyme inhibitory potential of the plant samples using Ellman’s assay [34].

Plant samples were dissolved in few drops of methanol and further diluted in phosphate buffer (0.1 M) in different concentrations (125–1000 μg/mL). AChE (518 U/mg) and BChE (7–16 U/mg) were diluted in 0.1 M phosphate buffer (pH 8.0) until final concentrations of 0.03 U/mL (AChE) and 0.01 U/mL (BChE) was obtained. Solutions of DTNB (Sigma-Aldrich, Germany) 0.2273 mM, ATchI (Sigma-Aldrich, UK) 0.5 mM and BTchI (Sigma-Aldrich, Switzerland) 0.5 mM were prepared in distilled water and kept in the eppendorf in refrigerator (8 °C). For each assay, enzyme solution of 5 μL was added to the cuvette followed by plant samples (205 μL) and DTNB reagent (5 μL). The solution mixture was maintained at 30 °C for 15 min using water bath with subsequent addition of substrate solution (5 μL). A double beam spectrophotometer (Thermo electron corporation, USA) was used to measure the absorbance at 412 nm. Galanthamine (Sigma-Aldrich, France) was used as positive control [35]. The absorbance along with the reaction time was taken for 4 min at 30 °C. The experiment was performed in triplicate. The percent enzyme activity and enzyme inhibition by control and tested samples were calculated from the rate of absorption with change in time (V = ΔAbs/Δt) as follow

$$ \mathrm{Enzyme}\  \mathrm{inhibition}\ \left(\%\right)=100-\mathrm{percent}\  \mathrm{enzyme}\  \mathrm{activity} $$

$$ \mathrm{Enzyme}\  \mathrm{activity}\ \left(\%\right)=100\times \mathrm{V}/{\mathrm{V}}_{\mathrm{max}}\left(\mathrm{where}\ {\mathrm{V}}_{\mathrm{max}}\mathrm{is}\  \mathrm{enzyme}\  \mathrm{activity}\  \mathrm{in}\  \mathrm{the}\  \mathrm{absence}\  \mathrm{of}\  \mathrm{in}\mathrm{hibitor}\  \mathrm{drug}\right). $$

### Quantitative antioxidant assays

#### DPPH free radical scavenging assay

The free radical scavenging ability of Nm.Cr, subsequent fractions and Nm.Sp were tested using DPPH (Sigma Aldrich USA). Different concentrations (62.5–1000 μg/mL) of tested samples were prepared in methanol. In clean and labeled test tubes, 2 mL of DPPH solution (0.002% in methanol) was mixed with 2 mL of different concentrations of tested samples separately. The tubes were incubated at room temperature in dark for 30 min and the absorbance was measured at 517 nm using UV spectrophotometer [36]. All experiments were performed in triplicate using ascorbic acid as standard [37]. The percent scavenging activity of the tested samples was calculated using the formula,

$$ \mathrm{Scavenging}\  \mathrm{activity}\ \left(\%\right)=\left[\left(\mathrm{A}\hbox{--} \mathrm{B}\right)/\mathrm{A}\right]\times 100 $$

Where A is absorbance of DPPH and B is absorbance of DPPH plus tested samples combination.

#### ABTS free radical scavenging assay

ABTS (Sigma Aldrich USA) assay was carried out according to the method reported previously [38]. The assay is based on the capacity of antioxidants to scavenge ABTS radical cation causing a reduction in absorbance at 734 nm. The ABTS solution was prepared by mixing 7 mM ABTS and 2.45 mM potassium persulphate solutions (Riedel-de Haen Germany) and then incubated in the dark at room temperature for 16 h. Before the assay, the solution was diluted with methanol to give an absorbance of 0.706 ± 0.001 at 734 nm. Different concentrations (62.5–1000 μg/mL) of plant extracts were prepared in the methanol. ABTS solution (3 mL) was added to each concentration of tested samples and absorbance was measured for 6 min after 1 min incubation [39]. Experiment was carried out in triplicate. Ascorbic acid was taken as standard. The percent scavenging activity of the tested samples calculated using the formula,

Scavenging activity (%) = [(A – B) / A] × 100, where A is absorbance of ABTS and B is absorbance of ABTS and tested samples in combination.

### Total reducing power assay

Total reducing power of the Nm.Cr, its subsequent fractions and Cr.Sp was determined according to the previously reported method [40]. Briefly, each sample (62.5–1000 μg) was dissolved in 1 ml of distilled water to which was added 2.5 ml of a 0.2 M phosphate buffer (pH 6.6) and 2.5 ml of a 1% (*w*/*v*) solution of potassium ferricyanide. The mixture was incubated in a water bath at 50 °C for 20 min. Following this, 2.5 ml of a 10% (w/v) trichloroacetic acid solution was added and the mixture was then centrifuged at 1750×g for 10 min. A 2.5 ml aliquot of the upper layer was combined with 2.5 ml of distilled water and 0.5 ml of a 0.1% (w/v) solution of ferric chloride. Absorbance of the reaction mixture was read spectrophotometrically at 700 nm. Increased absorbance of the reaction mixture indicates greater reducing power. Mean values from three independent samples were calculated for each sample.

### Qualitative antioxidant assay

Qualitative assay of various samples of *N. micrantha* were performed by TLC method as previously described [41]. Chemical constituents of the extracts were analyzed by thin layer chromatography (TLC). The TLC plates were developed with three solvent systems i.e., ethyl acetate/methanol/water (EMW), chloroform/ethyl acetate/formic acid (CEF), benzene/ethanol/ammonium hydroxide (BEA). For the detection of antioxidant activity, chromatograms were sprayed with 0.2% DPPH and ABTS solutions, as indicators. The presence of antioxidant compounds were detected by yellow spots against a purple background on TLC plates sprayed for DPPH and Whitish yellow spots against bluish background for ABTS assay. The development of the chromatograms was carried out in a closed tank and the plates were dried in the fume hood.

### Statistical analysis

The extract concentrations providing 50% inhibition (IC_50_) were calculated from the graph of percent inhibition versus extract concentrations in solution, using Microsoft Excel program.

Tow way ANOVA followed Bonferroni multiple comparison tests were applied for the comparison of positive control and test groups. *P* values < 0.05 were considered statistically significant. GraphPad Prism was used to draw the graphs. IC_50_ values and mean ± SEM were calculated at 95% confidence intervals.

## Results

### GC/MS analysis

**Fig. 1:**
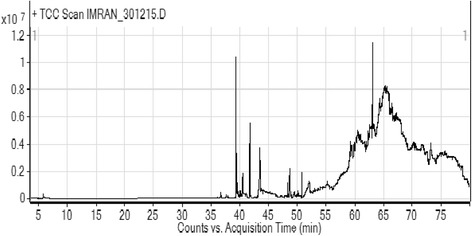
GC/Ms. spectra of Nm.Cr

The GC/MS analysis of Nm.Cr revealed different compounds as shown in Table 1 and Fig. 1. A total 37 compounds were present and identified. Some of the identified molecules include phytol, neophytadiene, decamethylene dibromide, crodacid, stigma-5-en-3-ol, methyl isoheptadecanoate, hexahydrofarnesyly acetone, pentadecyclic acid, chrysarobin, vanicol, myristaldehyde, methyl eicosanoate etc.

**Table 1:** List of compounds present Nm.Cr of *N.Micrantha* identified through GC/MS analysis

S.NO	Compound Label	RT	Common Name	Formula	Hits (DB)
1.	E-11,13-Tetradecadien-1-ol	3.769	NF	C14H26O	10
2.	Methane, sulfinylbis-	5.65	Dimethyl sulfoxide/ Hyadur	C2H6OS	10
3.	Tetradecylaldehyde	36.109	Myristaldehyde	C14H28O	10
4.	1,10-Dibromodecane	36.587	Decamethylene dibromide	C10H20Br2	10
5.	Tetradecanoic acid	37.593	Crodacid	C14H28O2	10
6.	(+)-.alpha.-Atlantone	37.793	(+)-.alpha.-Atlantone	C15H22O	1
7.	7,11,15-TRIMETHYL,3-METHYLENE-1-HEXADECENE	39.392	Neophytadiene	C20H38	10
8.	2-Pentadecanone, 6,10,14-trimethyl-	39.545	Hexahydrofarnesyl acetone	C18H36O	10
9.	7,11,15-TRIMETHYL,3-METHYLENE-1-HEXADECENE	40.028	Neophytadiene	C20H38	10
10.	Pentadecanoic acid	40.155	Pentadecylic acid	C15H30O2	10
11.	3,7,11,15-Tetramethyl-2-hexadecen-1-ol	40.485	NF	C20H40O	10
12.	Z-11-Hexadecenoic acid	41.601	NF	C16H30O2	10
13.	Hexadecanoic acid, methyl ester	41.734	Methyl palmitate / Uniphat A60	C17H34O2	10
14.	Hexadecanoic acid	43.481	Palmitinic acid / Prifrac 2960	C16H32O2	10
15.	1-phenylsulphonyl-1-trimethylsilylpropane	45.079	NF	C12H20O2SSi	3
16.	Hexadecanoic acid, 15-methyl-, methyl ester	45.243	Methyl isoheptadecanoate	C18H36O2	10
17.	4-Ethyloctanoic acid	46.188	NF	C10H20O2	2
18.	9,12-Octadecadienoic acid, methyl ester	48.274	NF	C19H34O2	10
19.	CYCLOOCTA-1,3-DIENE	48.632	NF	C8H12	10
20.	9-Octadecenoic acid (Z)-	48.945	methyl ester, Methyl oleate	C19H36O2	10
21.	2-Hexadecen-1-ol, 3,7,11,15-tetramethyl-, [R-[R*,R*-(E)]]-	49.426	Phytol	C20H40O	10
22.	Octadecanoic acid, methyl ester	50.107	Stearic acid, methyl ester	C19H38O2	10
23.	Tricyclo[4.3.1.0(2,5)]decane	52.065	NF	C10H16	10
24.	Oct-7-yn-1-ol	53.07	NF	C8H14O	1
25.	Octadecanoic acid	53.274	Vanicol	C18H36O2	10
26.	2-Methyltetradecanal	58.64	NF	C15H30O	1
27.	1,8,9-Anthracenetriol, 3-methyl-	58.759	Chrysarobin	C15H12O3	10
28.	Heptadecan-1-ol	59.016	1-Hydroxyheptadecane	C17H36O	10
29.	(1-Ethyloctyl)cyclohexane	59.265	NF	C16H32	10
30.	9,10-Anthracenedione, 1,8-dihydroxy-3-methyl-	59.468	C.I. Natural Yellow 23	C15H10O4	10
31.	Eicosanoic acid, methyl ester	59.719	Methyl eicosanoate	C21H42O2	10
32.	2H–Pyran-2-one, 6-heptyltetrahydro-	60.141	Delta.-Dodecalactone	C12H22O2	10
33.	3-Cyclopentylpropionic acid, 2-dimethylaminoethyl ester	61.94	NF	C12H23NO2	10
34.	D-Glucose, 2-O-[3-acetyl-1-(trimethylsilyl)-1H–indolyl]-3,4,5,6-tetrakis-O-(...	62.201	NF	C32H62N2O7Si5	1
35.	Docosanoic acid, methyl ester	62.904	Methyl behenate	C23H46O2	4
36.	1,2-Benzenedicarboxylic acid, bis(2-ethylhexyl) ester	63.069	DEHP / DNOP	C24H38O4	10
37.	Stigmast-5-en-3-ol, (3.beta.,24S)-	73.175	Clionasterol	C29H50O	2

### Acetylcholinesterase inhibitory activity

Among the tested fractions of *N. micrantha,* Nm.Hex and Nm.EtAc showed the strongest activity against AChE causing 75.51 ± 0.73 and 68.54 ± 0.59% inhibition respectively at 1000 μg/mL concentration (Table 2). All other fractions exhibited a dose dependent moderate inhibitory response. AChE inhibitory activity of different fractions were in order of Nm.Hex > Nm.EtAc > Nm.Cf > Nm.Aq > Nm.Cr > Nm.Sp. AChE inhibition by positive control galanthamine was 94.22 ± 1.01% at 1000 μg/mL and IC_50_ was <0.1 μg/ml.

**Table 2:** Percent anticholinesterase activity of various samples of *N. micrantha*

Sample	Concentration (μg/mL)	%AChE inhibition (mean ± SEM)	AChE IC_50_(μg/mL)	%BChE inhibition (mean ± SEM)	BChE IC_50_(μg/mL)
Nm.Cr	1000	54.32 ± 0.67***	340	75.31 ± 0.56***	44
	500	51.81 ± 0.52***		68.73 ± 0.43***	
	250	48.39 ± 0.59***		64.42 ± 0.76***	
	125	43.61 ± 0.32***		59.57 ± 0.38***	
Nm.Hex	1000	75.51 ± 0.73***	44	67.82 ± 0.34***	400
	500	69.67 ± 0.51***		53.44 ± 0.73***	
	250	64.35 ± 0.47***		39.53 ± 0.51***	
	125	58.66 ± 0.61***		30.67 ± 0.58***	
Nm.Cf	1000	63.48 ± 0.59***	144	53.76 ± 0.34***	750
	500	58.29 ± 0.83***		45.24 ± 0.61***	
	250	53.54 ± 0.52***		37.57 ± 0.83***	
	125	49.64 ± 0.44***		26.39 ± 0.42***	
Nm.EtAc	1000	68.54 ± 0.59***	100	54.71 ± 0.89***	720
	500	63.72 ± 0.34***		47.54 ± 0.44***	
	250	57.39 ± 0.78***		33.28 ± 0.76***	
	125	51.67 ± 0.63***		28.54 ± 1.22***	
Nm.Aq	1000	59.67 ± 0.57***	350	81.49 ± 0.89***	110
	500	53.72 ± 0.63***		76.52 ± 1.03***	
	250	46.34 ± 0.69***		58.39 ± 0.58***	
	125	41.58 ± 0.73***		53.47 ± 0.52***	
Nm.Sp	1000	47.56 ± 0.57***	1035	83.49 ± 0.27***	90
	500	44.31 ± 0.29***		71.52 ± 0.83***	
	250	38.73 ± 0.68***		66.41 ± 0.58***	
	125	31.59 ± 0.43***		53.73 ± 0.41***	
Galantamine	1000	94.22 ± 1.01	< 0.1	96.00 ± 0.30	< 0.1
	500	92.28 ± 0.43		92.90 ± 0.60	
	250	85.35 ± 0.83		89.45 ± 0.90	
	125	83.05 ± 1.02		86.23 ± 0.22	

Data is represented as mean ± SEM; *n* = 3. Values significantly different as compared to standared drug (Galanthamine) i.e. *: 0.05, **: 0.01 and ***: 0.001 at 90% confidence interval

### Butyrylcholinesterase inhibitory activity

Results of BChE inhibition assay are summarized in Table 2. In BChE inhibition assay, Nm.Sp, Nm.Aq and Nm.Cr fractions expressed the highest enzyme inhibition activity causing 83.49 ± 0.27, 81.49 ± 0.89 and 75.31 ± 0.56% inhibition at 1000 μg/mL concentration respectively. Median inhibitory concentrations (IC_50_) values for these fractions were 90, 110 and 44 μg/mL respectively. All other fractions showed inhibitory activity in concentration dependent manner. Percent BChE inhibitory activities of Nm.Sp (83.49 ± 0.27) and Nm.Aq (81.49 ± 0.89) were comparable to galantamine (96.00 ± 0.30) at 1000 μg/ml concentration whereas, IC_50_ of samples were quite high in comparison to control.

### Quantitative antioxidant assays

#### DPPH free radicals scavenging effect

**Fig. 2:**
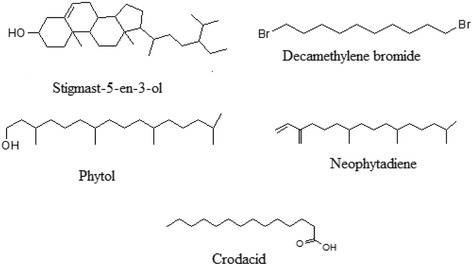
Percent DPPH scavenging activity of various samples of *Nonea micrantha*

DPPH free radical scavenging potentials of Nm.Cr, subsequent fractions and Nm.Sp of *N. micrantha* are summarized in Fig. 2. Among different fractions Nm.Aq, Nm.Hex and Nm.Cr expressed the highest antioxidant activity causing 88.25 ± 0.58, 84.56 ± 0.54 and 77.67 ± 0.53% inhibition of DPPH free radicals at 1000 μg/mL concentration respectively. IC_50_ values for these fractions were 3, 5 and 120 μg/ mL respectively (Fig. 4). Other fractions were also effective in concentration dependent pattern. DPPH free radical scavenging activities of different fractions were in descending order of Nm.Aq > Nm.Cf > Nm.Cr > Nm.Hex > Nm.EtAc > Nm.Sp. Ascorbic acid was used as positive control and its percent inhibition was 87.90 ± 0.96 at 1000 μg/mL concentration.

#### ABTS free radicals scavenging effect

**Fig. 3:**
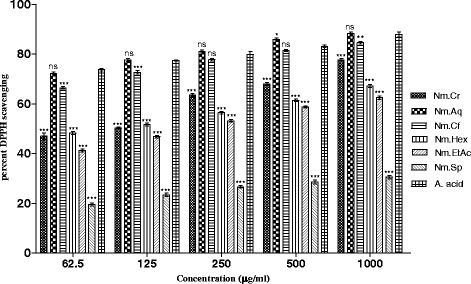
Percent ABTS scavenging activity of various samples of *Nonea micrantha*

**Fig. 4:**
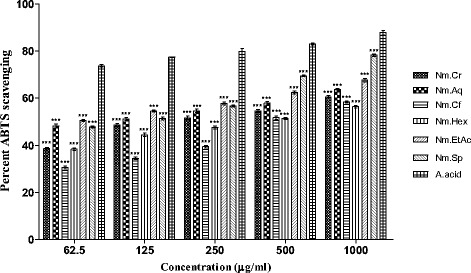
IC_50_ values for different fractions of *N. micrantha* in DPPH and ABTS assays

Results of ABTS free radical scavenging activities are given in Fig. 3. Highest ABTS free radical scavenging activity was observed for Nm.Sp, Nm.EtAc and Nm.Aq causing 78.26 ± 0.49, 67.67 ± 0.73 and 63.58 ± 0.45% inhibition at 1000 μg/mL concentration respectively as shown in Fig. 3. In comparison to DPPH scavenging assay, IC_50_ values were high for these fractions i.e. Nm.EtAc (60), Nm.Aq (95) and Nm.Sp (100) μg/ml (Fig. 4). Other fractions showed from moderate to good percent scavenging activity in concentration dependent manner. Ascorbic acid percent inhibition was 87.90 ± 0.96 at 1000 μg/mL concentration. Percent ABTS scavenging activity of Nm.Sp (78.26 ± 0.49) was comparable to ascorbic acid activity (87.90 ± 0.96) at the same concentration (1000 μg/ml).

### Total reducing power assay

**Fig. 5:**
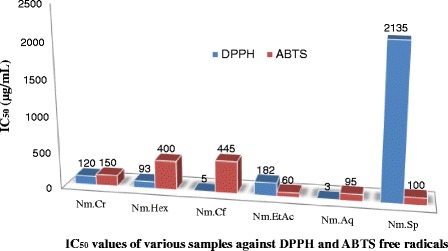
Results of Total reducing power (TRP) assay

As obvious from the Fig. 5, the reducing powers of the Nm.Cr, its subsequent fractions and Nm.Sp of *N. micrantha* in a concentration dependent manner. Among all the samples, Nm.Cf showed the highest reducing powers with 0.35 ± 0.01, 0.48 ± 0.03, 0.57 ± 0.02, 0.66 ± 0.03 and 0.76 ± 0.04 absorbance units at 62.5 to 1000 μg/mL concentrations respectively. This was followed by Nm.Aq and Nm.Cr showing 0.72 ± 0.03 and 0.71 ± 0.04 absorbance units at 1000 μg/mL concentration respectively. All the samples showed total reducing powers in concentration dependent manner in a descending order of Nm.Cf > Nm.Aq > Nm.Cr > Nm.EtAc > Nm.Sp > Nm.Hex. The results of Nm.Cf, Nm.Aq and Nm.Cr were quite comparable with those of the standard.

### Qualitative antioxidant assays

**Fig. 6:**
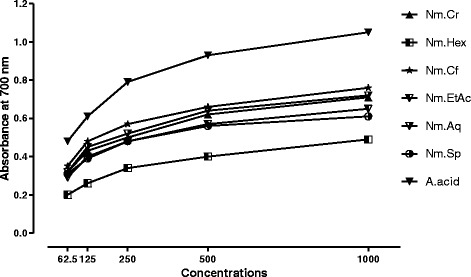
Results of qualitative antioxidant (DPPH) assay

**Fig. 7:**
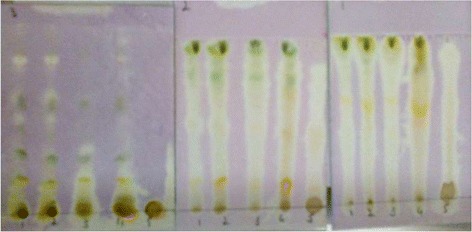
Results of qualitative antioxidant (ABTS) assay

Results of qualitative antioxidant assays are shown in Figs. 6 and 7. All fractions exhibited DPPH and ABTS scavenging activities as indicated by yellow spots against purple and bluish background respectively. In DPPH assay, Nm.Cr, Nm.Cf, Nm.Hex and Nm.EtAc showed that these fractions are enriched with antioxidant compounds. Likewise, in ABTS assay, results of TLC plates demonstrate that Nm.Cf, Nm.EtAc and Nm.Hex contain high concentrations of antioxidant compounds. Results are more prominent in CEF solvent system.

## Discussion

**Fig. 8:**
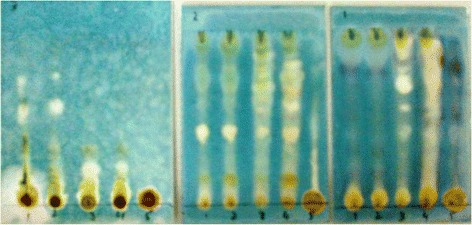
Bioactive compounds present in Nm.Cr

The GC/MS analysis revealed the presence of bioactive molecules in the crude extract of the plant as shown in Table 1 and Fig. 8. Molecules like phytol, neophytadiene, decamethylene dibromide, crodacid, stigma-5-en-3-ol have been reported for various pharmacological potentials. Phytol has been reported for anti-inflammatory and leukocyte recruitment potentials and cytokinene and oxidative stress inhibition activities. It has also been reported for anxiolytic activity and anti-convulsant potentials in animal’s model [42–44]. Similarly, neophytadiene has been reported for anti-inflammatory activity [45]. Decamethylene dibromide has been excellent molecule in respect of neuromuscular blocking activity [46]. Crodacid and stigma-5-en-3-ol have been found useful pharmaceutical molecules for antioxidant, anti-diabetic, anti-ulcer and anti-microbial activities [47–49].

Patients suffering from AD experience a loss of cholinergic synapses in the brain regions which are linked with higher intellectual functions. In AD patients, it is considered that a decrease in the ACh appears to be major element in the development of dementia. Hence, AD and other form of dementia could be treated by the use of agents that restore the level of acetylcholine through the inhibition of both major form of cholinesterase enzymes AChE and BChE [50]. The history of finding new drug candidates shows that plants are the major source for novel and active compounds and have become a priority of modern pharmaceutical industries. Many synthetic drugs owe their origin to plant based complementary medicine. Since AD, one of the most common causes of death worldwide has become a threat to public health, therefore, new treatment strategies based on medicinal plants are gaining attention [51].

From the literature survey of various medicinal plants it is obviously clear that plants possess certain secondary metabolites which are responsible for specific pharmacological activities. The most commonly reported group of secondary metabolite responsible for antioxidant activity is flavonoids. The flavonoids possess phenolic functional groups which have the ability to quench free radicals and exhibit miraculous antioxidant activity [34]. Similarly the saponins which are also considered as a very important group of secondary metabolite and have been reported to possess antioxidant as well as anticholinesterase activity [28]. The anticholinesterase activity of saponins isolated from *Xerospermum noronhianum* has been reported recently with good results [52]. Likewise in the current study it has been demonstrated that the saponins present in *N. micrantha* possess good antioxidant and anticholinesterase potentials. Moreover various active compounds and extracts obtained from medicinal plants such as *Gingko biloba, Huperzia serrata, Galanthus nivalis* and *Salvia officinalis* have been evaluated for their efficacy against AD, showing remarkable results [53]. Recently, *Garcinia combogia* was investigated for anticholinestrase activity in-vitro and a significant correlation between the total phenolic contents and anticholinestrase activity was established [54]. The ethanolic extract of *Bacopa monnieri* was investigated in-vivo on AChE activity in brain, demonstrated the highest AChE inhibition [55]. Withanolides, a group of naturally occurring steroids, have been isolated from the whole plant of *Withania somnifera* and were screened for the AChE and BChE inhibition potentials with good results [56]. In another study, AChE and BChE inhibitory activities of 19 essential oils obtained from cultivated plants have been reported with very high inhibitory potentials against both enzymes [57].

In our current study *N. micrantha* was investigated for AChE and BChE for the very first time for AChE inhibitory assay. It was revealed that Nm.Hex having the strongest activity causing 75.51 ± 0.73% inhibition at 1000 μg/mL concentration in comparison with standard galanthamine. Other fractions effective against AChE in dose dependent manner were Nm.EtAc and Nm.Cf with percent inhibition of 68.54 ± 0.59 and 63.48 ± 0.59 respectively at concentration of 1000 μg/mL. All other fractions i.e. Nm.Cr, Nm.Aq and Nm.Sp showed from moderate to good AChE inhibitory activity (Table 2). In BChE inhibition assay, Nm.Sp showed the strongest activity with percent inhibition of 83.49 ± 0.27 at 1000 μg/mL concentration. BChE inhibitory activity of Nm.Sp was comparable to positive control. Enzyme inhibition for other fractions including Nm.Aq and Nm.Cr were 81.49 ± 0.89 and 75.31 ± 0.56% at 1000 μg/mL respectively. Other fractions including Nm.Hex, Nm.Cf and Nm.EtAc also expressed moderate inhibitory results against BChE in comparison to the standard galanthamine (Table 2).

Free radicals are produced inside the body and mostly found in deep fried foods with spices. They are responsible for oxidation of cell lipids and DNA damage [58]. Presently available synthetic antioxidants like butylated hydroxy anisole, butylated hydroxy toluene, tertiary butylated hydroquinon and gallic acid esters have been suspected to cause negative health effects. Hence, a strong restriction has been placed on their applications and there is a trend to replace them with naturally occurring antioxidants with high efficacy and safety levels. Additionally these existing synthetic antioxidants are less soluble and show moderate antioxidant activity [59, 60]. Recently, there have been increased interests in the therapeutic potentials of medicinal plants as antioxidants in reducing such free radical induced tissue injuries. Beside this, well known and traditionally used natural antioxidants from tea, wine, fruits, vegetables, spices and some natural antioxidant such as rosemary and sage have already been exploited commercially either as antioxidant additives or a nutritional supplements [61].

In the current study antioxidant activity of plant samples has been evaluated in order to prove its effectiveness in neurological disorders. In DPPH and ABTS free radical scavenging (quantitative) methods Nm.Aq, Nm.Cf and Nm.Cr showed highest activity against DPPH free radicals causing 88.25 ± 0.58, 84.56 ± 0.54 and 77.67 ± 0.53% inhibition respectively at 1000 μg/ml concentration. These fractions were most potent as indicated by their IC_50_ values (Fig. 4). Similarly Nm.Sp, Nm.EtAc and Nm.Aq also showed good results against ABTS free radicals with 78.26 ± 0.49, 67.67 ± 0.73 and 63.58 ± 0.45 respectively in a concentration of 1000 μg/mL. Qualitative assay has confirmed the scavenging potential (Yellow color spots) of different fractions as shown in Figs. 6 and 7.

## Conclusion

Based on our current findings, it is concluded that *N. micrantha* is rich in bioactive compounds and a potential source of antioxidant and anticholinesterase compounds. Similarly, the significant results demonstrated by saponins isolated from *N. micrantha* reveals that Nm.Sp should be further purified, characterized and should be the part of complementary and alternative medicine. It is further needed to isolate and investigate the essentials oils, secondary metabolites and other bioactive compounds from this plant having potential to combat the diseases such as AD, Parkinsonism, amnesia and dementias.

## Abbreviations

ABTS: 2,2-Azinobis[3-ethylbenzthiazoine]-6-sulfonic acid
AChE: Acetylcholinesterase
AD: Alzheimer’s disease
AIDS: Acquired immunodeficiency syndrome
BChE: Butyrylcholinesterase
BHA: Butylated hyroxyanisole
BHT: Butylated hydroxytoluene
DPPH: 1,1-Diphenyl 2-picrylhydrazyl radical
DTNB: (5,5′-dithio-bis-[2-nitrobenzoic acid])
GC-MS: Gas chromatography- mass spectrometry
IC_50_: Median inhibitory concentration
*N. micrantha*: *Nonea micrantha*
NaCl: Sodium chloride
Nm.Aq: Aqueous fraction of *Nonea micrantha*
Nm.Cf: Chloroform fraction of *Nonea micrantha*
Nm.Cr: Crude methanolic extract of *Nonea micrantha*
Nm.EtAc: Ethyl acetate fraction of *Nonea micrantha*
Nm.Hex: *n*-hexane fraction of *Nonea micrantha*
Nm.Sp: Crude saponins fraction of *Nonea micrantha*
ROS: Reactive oxygen species
TBHQ: Tertiary butyl hydroquinone
TLC: Thin layer chromatography
TRP: Total reducing power

## References

[1] Ferri CP, Prince M, Brayne C, Brodaty H, Fratiglioni L, Ganguli M, Hall K, Hasegawa K, Hendrie H, Huang Y (2006). Global prevalence of dementia: a Delphi consensus study. Lancet.

[2] Bachman D, Wolf PA, Linn R, Knoefel J, CobbS J, Belanger A, D'Agostino R, White L (1992). Prevalence of dementia and probable senile dementia of the Alzheimer type in the Framingham study. Neurology.

[3] Ayaz M, Junaid M, Ullah F, Subhan F, Sadiq A, Ali G, Ovais M, Shahid M, Ahmad A, Wadood A, et al. Anti-Alzheimer’s studies on beta-Sitosterol isolated from *Polygonum hydropiper* L. Front Pharmacol. 2017;8:697. doi:10.3389/fphar.2017.00697. 10.3389/fphar.2017.00697 PMC5635809 29056913

[4] Hebert LE, Scherr PA, Beckett LA, Albert MS, Pilgrim DM, Chown MJ, Funkenstein HH, Evans DA (1995). Age-specific incidence of Alzheimer's disease in a community population. JAMA.

[5] Ali M, Muhammad S, Shah MR, Khan A, Rashid U, Farooq U, Ullah F, Sadiq A, Ayaz M, Ali M (2017). Neurologically potent molecules from Crataegus Oxyacantha; isolation, Anticholinesterase inhibition, and molecular docking. Front Pharmacol.

[6] Sung SH, Kang SY, Lee KY, Park MJ, Kim JH, Park JH, Kim YC, Kim J, Kim YC (2002). **(+)-α-viniferin, a stilbene trimer from Caragana chamlague, inhibits acetylcholinesterase**. Biol Pharm Bull.

[7] Ayaz M, Junaid M, Ullah F, Sadiq A, Khan MA, Ahmad W, Shah MR, Imran M, Ahmad S (2015). Comparative chemical profiling, cholinesterase inhibitions and anti-radicals properties of essential oils from Polygonum Hydropiper L: a preliminary anti-Alzheimer's study. Lipids Health Dis.

[8] Ayaz M, Sadiq A, Junaid M, Ullah F, Subhan F, Ahmed J (2017). Neuroprotective and anti-aging potentials of essential oils from aromatic and medicinal plants. Front Aging Neurosci.

[9] Gülçin İ, Oktay M, Küfrevioğlu Öİ, Aslan A (2002). Determination of antioxidant activity of lichen Cetraria Islandica (L) ach. J Ethnopharmacol.

[10] Ullah F, Ayaz M, Sadiq A, Hussain A, Ahmad S, Imran M, Zeb A (2016). Phenolic, flavonoid contents, anticholinesterase and antioxidant evaluation of Iris Germanica var; florentina. Nat Prod Res.

[11] Cook N, Samman S (1996). Flavonoids—chemistry, metabolism, cardioprotective effects, and dietary sources. J Nutr Biochem.

[12] Sies H (1997). Oxidative stress: oxidants and antioxidants. Exp Physiol.

[13] Shasha D, Magogo C, Dzomba P (2014). Reversed phase HPLC-UV Quantitation of BHA, BHT and TBHQ in food items sold in Bindura supermarkets, Zimbabwe. Int Res J Pure Appl Chem.

[14] Ahmad S, Ullah F, Sadiq A, Ayaz M, Imran M, Ali I, Zeb A, Ullah F, Shah MR (2016). Chemical composition, antioxidant and anticholinesterase potentials of essential oil of Rumex Hastatus D. Don collected from the north west of Pakistan. BMC Complement Altern Med.

[15] Sadiq A, Mahmood F, Ullah F, Ayaz M, Ahmad S, Haq FU, Khan G, Jan MS (2015). Synthesis, anticholinesterase and antioxidant potentials of ketoesters derivatives of succinimides: a possible role in the management of Alzheimer’s. Chem Cent J.

[16] Shu Y-Z (1998). Recent natural products based drug development: a pharmaceutical industry perspective. J Nat Prod.

[17] Cai Y, Sun M, Corke H (2003). Antioxidant activity of betalains from plants of the Amaranthaceae. J Agric Food Chem.

[18] Fusco D, Colloca G, Monaco MRL, Cesari M (2007). Effects of antioxidant supplementation on the aging process. Clin Interv Aging.

[19] Amoo SO, Aremu AO, Moyo M, Van Staden J (2012). Antioxidant and acetylcholinesterase-inhibitory properties of long-term stored medicinal plants. BMC Complement Altern Med.

[20] Shah S, Shah SMM, Ahmad Z, Yaseen M, Shah R, Sadiq A, Khan S, Khan B: Phytochemicals, in vitro antioxidant, total phenolic contents and phytotoxic activity of Cornus Macrophylla wall bark collected from the north-west of Pakistan. Pak J Pharm Sci 2015, 28(1):23-28. 25553682

[21] Shah A, Marwat SK, Gohar F, Khan A, Bhatti KH, Amin M, Din NU, Ahmad M, Zafar M. Ethnobotanical study of medicinal plants of semi-tribal area of Makerwal & Gulla Khel (lying between Khyber Pakhtunkhwa and Punjab provinces), Pakistan. Am J Plant Sci. 2013;4(1):98-116.

[22] Di Stasi L, Oliveira G, Carvalhaes M, Queiroz-Junior M, Tien O, Kakinami S, Reis M (2002). Medicinal plants popularly used in the Brazilian tropical Atlantic Forest. Fitoterapia.

[23] Okusa P, Penge O, Devleeschouwer M, Duez P (2007). Direct and indirect antimicrobial effects and antioxidant activity of< i> Cordia gilletii</i> de wild (< i> Boraginaceae</i>). J Ethnopharmacol.

[24] Kelley CJ, Harruff RC, Carmack M (1976). Polyphenolic acids of Lithospermum Ruderale. II. Carbon-13 nuclear magnetic resonance of lithospermic and rosmarinic acids. J Org Chem.

[25] Rice-evans CA, Miller NJ, Bolwell PG, Bramley PM, Pridham JB (1995). The relative antioxidant activities of plant-derived polyphenolic flavonoids. Free Radic Res.

[26] Ahmad S, Ullah F, Ayaz M, Sadiq A, Imran M (2015). Antioxidant and anticholinesterase investigations of Rumex Hastatus D. Don: potential effectiveness in oxidative stress and neurological disorders. Biol Res.

[27] Ayaz M, Junaid M, Ullah F, Sadiq A, Subhan F, Khan MA, Ahmad W, Ali G, Imran M, Ahmad S (2016). Molecularly characterized solvent extracts and saponins from *Polygonum hydropiper* L show high anti-angiogenic, anti-tumor, brine shrimp and fibroblast NIH/3T3 cell line cytotoxicity. Front Pharmacol.

[28] Ayaz M, Junaid M, Ahmed J, Ullah F, Sadiq A, Ahmad S, Imran M (2014). Phenolic contents, antioxidant and anticholinesterase potentials of crude extract, subsequent fractions and crude saponins from Polygonum Hydropiper L. BMC Complement Altern Med.

[29] Zeb A, Sadiq A, Ullah F, Ahmad S, Ayaz M (2014). Phytochemical and toxicological investigations of crude methanolic extracts, subsequent fractions and crude saponins of *Isodon rugosus*. Biol Res.

[30] Shah SMM, Sadiq A, Shah SMH, Khan S (2014). Extraction of saponins and toxicological profile of Teucrium stocksianum boiss extracts collected from district swat, Pakistan. Biol Res.

[31] Kamal Z, Ullah F, Ahmad S, Ayaz M, Sadiq A, Imran M, Ahmad S, Rahman FU, Zeb A (2017). Saponins and solvent extracts from Atriplex Laciniata L. exhibited high anthelmintic and insecticidal activities. J Tradit Chin Med.

[32] Ayaz M, Junaid M, Ullah F, Sadiq A, Ovais M, Ahmad W, Zeb A (2016). Chemical profiling, antimicrobial and insecticidal evaluations of Polygonum Hydropiper L. BMC Complement Altern Med.

[33] Ayaz M, Junaid M, Ullah F, Sadiq A, Shahid M, Ahmad W, Ullah I, Ahmad A (2017). Syed N-i-H: **GC-MS analysis and Gastroprotective evaluations of crude extracts, isolated Saponins, and essential oil from Polygonum Hydropiper L**. Front Chem.

[34] Kamal Z, Ullah F, Ayaz M, Sadiq A, Ahmad S, Zeb A, Hussain A, Imran M (2015). Anticholinesterse and antioxidant investigations of crude extracts, subsequent fractions, saponins and flavonoids of atriplex laciniata L.: potential effectiveness in Alzheimer's and other neurological disorders. Biol Res.

[35] Zeb A, Sadiq A, Ullah F, Ahmad S, Ayaz M (2014). Investigations of anticholinesterase and antioxidant potentials of methanolic extract, subsequent fractions, crude saponins and flavonoids isolated from *Isodon rugosus*. Biol Res.

[36] Shah SM, Ayaz M, Khan A-u, Ullah F, Farhan, Shah A-u-HA, Iqbal H, Hussain S (2015). **1,1-Diphenyl,2-picrylhydrazyl free radical scavenging, bactericidal, fungicidal and leishmanicidal properties of Teucrium stocksianum**. Toxicol Ind Health.

[37] Kekuda T, Shobha K, Onkarappa R (2010). Studies on antioxidant and anthelmintic activity of two Streptomyces species isolated from western Ghat soils of Agumbe, Karnataka. J Pharm Res..

[38] Re R, Pellegrini N, Proteggente A, Pannala A, Yang M, Rice-Evans C (1999). Antioxidant activity applying an improved ABTS radical cation decolorization assay. Free Radic Biol Med.

[39] Ullah F, Iqbal N, Ayaz M, Sadiq A, Ullah I, Ahmad S, Imran M. DPPH, ABTS free radical scavenging, antibacterial and phytochemical evaluation of crude methanolic extract and subsequent fractions of Chenopodium Botrys aerial parts. Pak J Pharm Sci. 2017;30(3) 28653919

[40] Benzie IF, Strain JJ (1996). The ferric reducing ability of plasma (FRAP) as a measure of “antioxidant power”: the FRAP assay. Anal Biochem.

[41] Masoko E (2007). Screening of twenty-four south african *combretum* and six *terminalia* species (combretaceae) for antioxidant activities. Afr J Trad Cam.

[42] Costa JP, de Oliveira GAL, de Almeida AAC, Islam MT, de Sousa DP, de Freitas RM (2014). Anxiolytic-like effects of phytol: possible involvement of GABAergic transmission. Brain Res.

[43] Silva RO, Sousa FBM, Damasceno SR, Carvalho NS, Silva VG, Oliveira FR, Sousa DP, Aragão KS, Barbosa AL, Freitas RM (2014). Phytol, a diterpene alcohol, inhibits the inflammatory response by reducing cytokine production and oxidative stress. Fundam Clin Pharmacol.

[44] Costa J, Ferreira P, De Sousa D, Jordan J, Freitas R (2012). Anticonvulsant effect of phytol in a pilocarpine model in mice. Neurosci Lett.

[45] Carretero M, López-Pérez J, Abad M, Bermejo P, Tillet S, Israel A, Noguera-P B (2008). Preliminary study of the anti-inflammatory activity of hexane extract and fractions from Bursera Simaruba (Linneo) Sarg.(Burseraceae) leaves. J Ethnopharmacol.

[46] Taylor E. 258. Synthetic neuromuscular blocking agents. Part I. Heterocyclic decamethylenebis (quaternary ammonium salts). J Chem Soc (Resumed). 1951:1150–7.

[47] Zitterl-Eglseer K, Sosa S, Jurenitsch J, Schubert-Zsilavecz M, Della Loggia R, Tubaro A, Bertoldi M, Franz C (1997). Anti-oedematous activities of the main triterpendiol esters of marigold (Calendula Officinalis L.). J Ethnopharmacol.

[48] Sujatha S, Anand S, Sangeetha K, Shilpa K, Lakshmi J, Balakrishnan A, Lakshmi B (2010). Biological evaluation of (3β)-STIGMAST-5-EN-3-OL as potent anti-diabetic agent in regulating glucose transport using in vitro model. Int J Diab Mellitus.

[49] Zhu M, Lew KT, Leung P (2002). Protective effect of a plant formula on ethanol-induced gastric lesions in rats. Phytother Res.

[50] Loizzo MR, Tundis R, Menichini F, Menichini F (2008). Natural products and their derivatives as cholinesterase inhibitors in the treatment of neurodegenerative disorders: an update. Curr Med Chem.

[51] Howes MJR, Perry NS, Houghton PJ (2003). Plants with traditional uses and activities, relevant to the management of Alzheimer's disease and other cognitive disorders. Phytother Res.

[52] Jean TP, Shaari K, Paetz C, Ismail IS, Abas F, Lajis NH, Ahmad VU (2009). Bidesmosidic oleanane saponins from Xerospermum noronhianum. Helv Chim Acta.

[53] Mantle D, Pickering AT, Perry EK (2000). Medicinal plant extracts for the treatment of dementia. CNS Drugs.

[54] Subhashini N, Nagarajan G, Kavimani S (2011). In vitro antioxidant and anticholinesterase activities of Garcinia combogia. Int J Pharm Pharm Sci.

[55] Ahirwar S, Tembhre M, Gour S, Namdeo A (2012). Anticholinesterase efficacy of Bacopa Monnieri against the brain regions of rat-a novel approach to therapy for Alzheimer’s disease. Asian J Exp Sci.

[56] Choudhary MI, Yousuf S, Nawaz SA, Ahmed S (2004). Cholinesterase inhibiting withanolides from Withania Somnifera. Chem Pharm Bull.

[57] Orhan I, Kartal M, Kan Y, Sener B (2008). Activity of essential oils and individual components against acetyl-and butyrylcholinesterase. Z Naturforsch C.

[58] Asokkumar K, Umamaheswari M, Sivashanmugam A, Subhadradevi V, Subhashini N, Ravi T (2008). Antioxidant activities of Erythrina Stricta Roxb. Using various in vitro and ex vivo models. Oriental Pharm Exp Med.

[59] Barlow SM. Toxicological aspects of antioxidants used as food additives. In: Hudson BJF, editors. Food antioxidants. Elsevier Applied Food Science Series. Dordrecht: Springer; 1990. p. 253–307.

[60] Branen A (1975). Toxicology and biochemistry of butylated hydroxyanisole and butylated hydroxytoluene. J Am Oil Chem Soc.

[61] Schuler P. Natural antioxidants exploited commercially. In: Hudson BJF, editor. Food antioxidants. Elsevier Applied Food Science Series. Dordrecht: Springer; 1990. p. 99–170.

